# Optical vortices 30 years on: OAM manipulation from topological charge to multiple singularities

**DOI:** 10.1038/s41377-019-0194-2

**Published:** 2019-10-02

**Authors:** Yijie Shen, Xuejiao Wang, Zhenwei Xie, Changjun Min, Xing Fu, Qiang Liu, Mali Gong, Xiaocong Yuan

**Affiliations:** 1 Key Laboratory of Photonic Control Technology (Tsinghua University), Ministry of Education, 100084 Beijing, China; 2 State Key Laboratory of Precision Measurement Technology and Instruments, Department of Precision Instrument, Tsinghua University, 100084 Beijing, China; 3 National Engineering Laboratory for Public Safety Risk Perception and Control by Big Data (NEL-PSRPC), China Academy of Electronics and Information Technology of CETC, China Electronic Technology Group Corporation, 100041 Beijing, China; 4 Nanophotonics Research Center, Shenzhen University, 518060 Shenzhen, China

**Keywords:** Optical physics, Optical physics, Optical physics, Optical physics

## Abstract

Thirty years ago, Coullet et al. proposed that a special optical field exists in laser cavities bearing some analogy with the superfluid vortex. Since then, optical vortices have been widely studied, inspired by the hydrodynamics sharing similar mathematics. Akin to a fluid vortex with a central flow singularity, an optical vortex beam has a phase singularity with a certain topological charge, giving rise to a hollow intensity distribution. Such a beam with helical phase fronts and orbital angular momentum reveals a subtle connection between macroscopic physical optics and microscopic quantum optics. These amazing properties provide a new understanding of a wide range of optical and physical phenomena, including twisting photons, spin–orbital interactions, Bose–Einstein condensates, etc., while the associated technologies for manipulating optical vortices have become increasingly tunable and flexible. Hitherto, owing to these salient properties and optical manipulation technologies, tunable vortex beams have engendered tremendous advanced applications such as optical tweezers, high-order quantum entanglement, and nonlinear optics. This article reviews the recent progress in tunable vortex technologies along with their advanced applications.

## Optical vortices: looking back at twisted light

Commemorating the 30th anniversary of the prediction of optical vortices by theoretical physicist Pierre Coullet and colleagues, researchers in China review the development of understanding and applications of these intriguing phenomena. Xing Fu at Tsinghua University, Xiaocong Yuan at Shenzhen University and co-authors, explain how the concept of optical vortices emerged from observed similarities between the behaviour of fluid vortices and some forms of laser light. The light waves of optical vortices are twisted around their direction of travel, with a point of zero intensity at their centre. The authors survey the steady refinement of techniques used to create optical vortices, and explore their applications. Prominent applications include sophisticated optical computing processes, novel microscopy and imaging techniques, the creation of ‘optical tweezers’ to trap particles of matter, and optical machining using light to pattern structures on the nanoscale.

## Introduction

Vortices are common phenomena that widely exist in nature, from quantum vortices in liquid nitrogen to ocean circulation and typhoon vortices and even to spiral galaxies in the Milky Way, manifesting themselves not only in macroscopic matter but also in structured electromagnetic and optical fields. This year is the 30th anniversary of the birth of optical vortices (OVs). In 1989, Coullet et al.^1^ found the vortex solutions of the Maxwell-Bloch equations and created the concept of OVs, inspired by hydrodynamic vortices. Before the proposal of OVs, the analogy between laser physics and fluids/superfluids was already recognized^2^ as early as 1970 by reducing the laser equations to complex Ginzburg–Landau equations (CGLEs), which constitute a class of universal models describing pattern formation in a vast variety of phenomena such as superconductivity, superfluidity, and Bose-Einstein condensation^3^. Later, many hydrodynamic features, such as chaos, multistability, and turbulence, were analogically studied in optical fields^4–6^ and observed in laser systems^7–9^. Among the various hydrodynamic effects, the vortex soliton is quite attractive due to its distinctive structure carrying a singularity^5–7^. Analogous to the flow singularity in a fluid vortex, an optical vortex soliton has a phase singularity that appears as an isolated dark spot possessing the topological charges (TCs) of a helical phase^5,10^. Novel optical vortex solitons were intensively explored based on CGLEs. For instance, stable vortex solitons^11^ and dissipative vortex solitons with trapping potentials^12^ can be solved by two-dimensional CGLEs. Topologically multicharged rotating vortex solitons^13^ and vortex excitation with feedback^14^ in lasers were also studied by CGLEs. Moreover, complicated three-dimensional toroidal dissipative vortex solitons^15^ can also be characterized by CGLEs with high-order nonlinearity. In 1992, Allen et al.^16^ proposed the orbital angular momentum (OAM) in vortex beams (VBs) where the OVs propagate in paraxial beams, which unveiled a new understanding of the connection between macroscopic optics and quantum effects. As a typical representative of OVs, a VB has become a classical tool to study the properties of OVs because its generation can be easily realized in the laboratory^17^. VBs characterized by Hilbert factor exp(i*ℓθ*), e.g., the Laguerre–Gaussian (LG) modes, can carry OAM equivalent to *ℓ*ℏ per photon (*ℓ* is an integer number), and this angular momentum (AM) can be much greater than the spin angular momentum (SAM) related to the photon spin^10^. The general results of these investigations created a new chapter of modern optics, i.e., singular optics^18^, which is a great leap forward in the development of traditional optics.

In the first 10 years, 1989–1999, the studies on OVs mainly focused on establishing fundamental theories and exploring basic physical phenomena, paving the way for further studies of the light–matter interaction, topological structures, and quantum nature of light. For instance, the dynamics of transverse pattern formation^5,6^, the interaction and OAM transfer between OVs and particles^19–21^, vortex solitons in a nonlinear medium^22–24^, nonlinear OAM-frequency transformation^25,26^, the topological phase in OVs^27^, the rotational Doppler effect^28^, and multi-singularity arrays or vortex crystals^5,29^ were thoroughly studied. These novel theories lay the foundation for extending further widespread applications by using the unique properties of OVs.

In the second 10 years, 1999–2009, with the development of OAM manipulation, tremendous new applications rapidly emerged. In 2001, Zeilinger’s group^30^ realized the OAM-entangled photon pair, bringing OVs or twisted photons into quantum applications^31^. In 2002, Dholakia’s group trapped particles with controlled rotation^32^ and a three-dimensional structure^33^ by VBs, expanding the applications of optical tweezers^34^. In 2003, Harwit^35^ demonstrated astrophysical OAM light generation and its applications in astrometry. In 2004, Zhuang^36^ used VBs as tweezers to assemble DNA biomolecules, opening up biomedical applications of OVs. In 2005, Ritsch-Marte’s group^37^ used OAM in microscopy and imaging, and Tamburini et al.^38^ reported a super-diffraction-limit imaging approach using OAM. In 2008, Barreiro et al.^39^ presented a coding technology using OAM, giving VBs great advantage for use in optical communications. During the decade, OVs were extended to almost every field of advanced optics.

In the last 10 years, 2009–2019, vortex and OAM applications have made many breakthroughs in rapid succession. In 2010, the optical lattice in far-field diffraction of OVs was unveiled as a very prompt and handy way to detect the TC^40^. In 2011, Capasso’s group^41^ proposed the generalized laws of reflection and refraction, guiding OV generation in nanoscale metasurfaces. In 2012, OAM beams were directly generated in a nanoscale emitter^42^. In 2013, Willner’s group demonstrated terabit-scale high-capacity optical communication via OAM multiplexing in both free space^43^ and fibres^44^. In 2016, Zeilinger’s group^45^ generated extreme OAM states of over 10,000ℏ and realized quantum entanglement of these states. In the recent three years, increasing numbers of tunable properties of OVs have been flexibly controlled at the nanoscale, including SAM–OAM conversion for classical^46^ and quantum light^47^, tunable wavelength from visible^48,49^ to X-ray light^50^, ultra-broadband tunable OAM^51^, and tunable chirality^52^. Moreover, the time-varying OAM was recently revealed in extreme-ultraviolet VB with time-delay-tunable high harmonic generation^53^. To date, OVs have brought about numerous innovations in various fields and are still enabling great novelties with improved tunability.

**Fig. 1:**
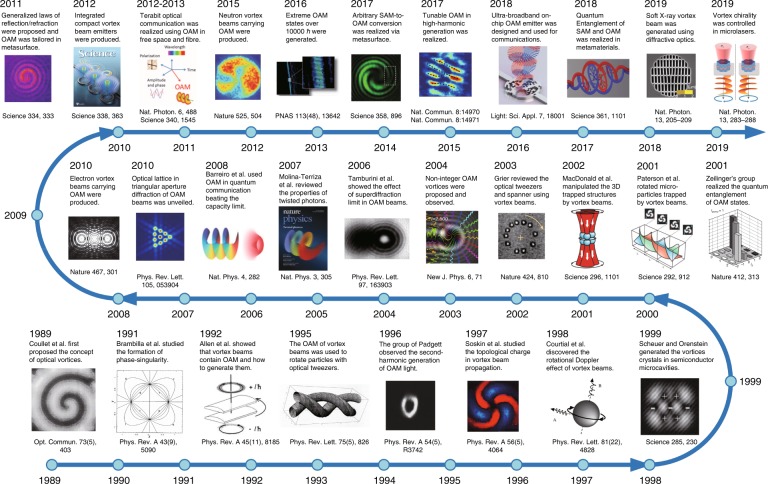
Roadmap of the 30-year development of optical vortices from 1989 to 2019, including significant theoretical and technical breakthroughs with corresponding references. Reprinted with permission from ref. ^1^, Copyright (2019), with permission from Elsevier. Reprinted with permission from refs. ^5,16,19,25,27,28,38,40^. Copyright (2019) by the American Physical Society. From refs. ^29,32,33,41,42,44,46,47^. Reprinted with permission from AAAS. Reprinted by permission from Springer Nature: Nature^30,34,59,60^, Nature Physics^31,39^, Nature Photonics^43,50,52^, Nature Communications^48,49^, Light: Science & Applications^51^, Copyright (2019). From ref. ^57^. Reproduced by permission of IOP Publishing

Throughout the roadmap summarized above and depicted in Fig. 1, we can divide the 30-year development into three stages: the first 10 years, the fundamental theories stage; the second, the application development stage; and the third, the technology breakthrough stage. These three stages share one common theme of pursuing improved tunability of OVs because the realization of a broader tunable range of OVs can always benefit the birth of new applications. Thus, we propose the tunability of OVs as a better way to describe state-of-the-art achievements of OVs. Traditionally, tunable light always means that the wavelength can be tuned and sometimes means that the pulse width can be tuned for a pulsed laser; however, the tunability of OVs should be expanded to more dimensions due to their exotic properties. The tunability of OVs includes not only the spectral and temporal tunability but also the OAM-, chirality-, TC-, and singularity-distribution tunability. We present reviews in succession on the historical progress in the new tunable methods of OVs driven by the fundamental theories and then the numerous novel applications engendered by the improved tunability of OVs. In the “Properties of OVs” section, we review the fundamental theories and properties of OVs, providing a better understanding of the corresponding applications enabled by the unique properties. In “Progress in vortex generation, tuning, and manipulation” section, we review the generation and manipulation methods of OVs, developed from tuning the TC of a single singularity to controlling a multi-singularity array, including wavelength-, temporal-, and OAM-tuning technologies. In “Advanced applications of tunable VBs” section, we comprehensively review various advanced applications derived from vortex manipulation. Concluding remarks and prospects are given in the “Conclusions and perspectives” section.

## Properties of OVs

### Singularity and topological charge

**Fig. 2:**
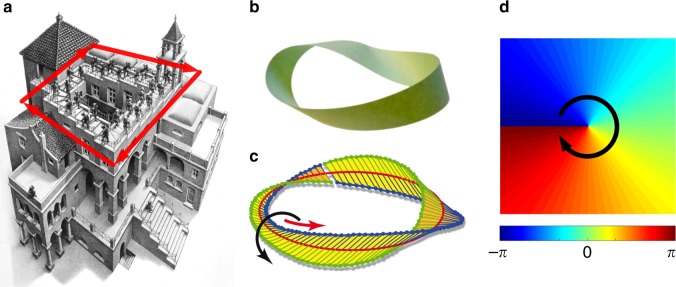
Basic topological structure of vortex from art to science. The topological structures of **a** the Penrose Stair^55^, **b**, **c** a Möbius strip^56^, and **d** the phase of a vortex soliton (Hilbert factor) are isomorphic, i.e., a physical value (displacement or angle) continually increases along a closed loop and coincides exactly with the origin after a roundtrip. **c** From ref. ^56^. Reprinted with permission from AAAS

The salient properties of OVs are mostly related to the topological phase structure. Early in the 1970s before OVs were first observed, the topological structure in the wave phase was already under study. Nye and Berry^54^ demonstrated that wave trains with dislocations could induce a vortex structure where a singularity could be solved in the wave equation, which laid the foundation for the study of vortices in air, water, and even light waves, pushing the discovery of OVs. To understand the profound topology in a plain way, we can refer to a familiar artwork exhibiting a similar structure. Escher’s painting Ascending and Descending shows an impossible scenario where the stairs are ascending clockwise yet have a seamless connection to their origin after a roundtrip, which is an artistic implementation of the Penrose Stairs^55^, as illustrated in Fig. 2. This structure is impossible in real space but possible in phase space. If the phase angle continually increases clockwise along a closed loop from 0 to 2*πℓ* and returns to the origin, where the integer *ℓ* is called the TC, the angle zero is exactly equal to 2*πℓ*, forming a continuous phase distribution along the closed loop, similar to the topology of the well-known Möbius strip^56^. The centre spot of the closed loop where the phase cannot be defined is a phase singularity. The definition of the TC of a singularity for the phase distribution $\varphi$ is given by:

1 $$\ell = \frac{1}{{2\pi }} \times {\oint}_C {\nabla \varphi \left( {\mathbf{r}} \right){\mathrm{dr}}}$$

where *C* is a tiny closed loop surrounding the singularity. For the light field with phase distribution exp(i*ℓθ*) carrying OAM of *ℓ*ℏ per photon, the TC of the centre phase singularity is *ℓ*. The effect of TC is actually commonly seen in our daily life, e.g., the time distribution on earth has a singularity at the North Pole with a TC of 24 h, the duration that the earth takes to rotate one cycle. The continuous phase along the closed loop results in an integer TC. However, as a peculiar case, a non-integer TC was also experimentally and theoretically investigated in OVs^57,58^. A phase singularity with a certain TC is a representation of a very simple vortex soliton yet acts as an important unit element in that more complex hydrodynamic vortices with chaos, attractors, and turbulence can be seen as the combination of a set of various singularities. This basic description is widely applicable to air^53^, water^4^, light^1^, electron^59^, and neutron^60^ vortex fields.

### Orbital angular momentum and vortex beams

A VB is a paraxial light beam possessing Hilbert factor exp(i*ℓθ*) and carrying OVs along the propagation axis. OVs are not restricted to VBs, yet as typical OVs, VBs carrying OAM, also called OAM beams, are almost the most attractive form of OVs due to their unique quantum-classical-connection properties. There are already many review articles on OAM, especially on vortex generation^61,62^, OAM on metasurfaces^63^, and basic OAM theories and applications^64,65^. However, few studies have focused on vortex tunability, which is the main theme of this article. For the introduction of basic theories of OAM, previous reviews usually used the well-known Poynting picture to describe the AM of the photon^66,67^, which leads to some difficulties, such as complex expressions of OAM and SAM, incompatibility with quantum optics, and the Abraham–Minkowski dilemma^68^. Here, we review the recently proposed new theory of the canonical picture^69,70^, which can overcome these difficulties, to introduce basic properties of OAM. The canonical momentum of light is represented as

2 $${\mathbf{P}} = \frac{g}{2}{\rm Im} \left[ {\tilde \varepsilon {\mathbf{E}}^ \ast \cdot \left( \nabla \right){\mathbf{E}} + \tilde \mu {\mathbf{H}}^ \ast \cdot \left( \nabla \right){\mathbf{H}}} \right]$$

where **H** is the magnetizing field. Gaussian units with $g = (8\pi \omega )^{ - 1}$, $\tilde \varepsilon = \varepsilon + \omega {\mathrm{d}}\varepsilon {\mathrm{/d}}\omega$, and $\tilde \mu = \mu + \omega {\mathrm{d}}\mu {\mathrm{/d}}\omega$ are used. The canonical SAM and OAM densities are expressed as

3 $${\mathbf{S}} = \frac{g}{2}{\rm Im} \left[ {\tilde \varepsilon {\mathbf{E}}^ \ast \times {\mathbf{E}} + \tilde \mu {\mathbf{H}}^ \ast \times {\mathbf{H}}} \right],{\mathbf{L}} = {\mathbf{r}} \;\times {\mathbf{P}}$$

The total AM of light is **J** = **S** + **L**. For a light beam, a rotating polarization leads to SAM, while a rotating wavefront leads to OAM. Consider a VB propagating along the *z*-axis:

4 $${\mathbf{E}}\left( {r,\theta ,z} \right) = A\left( {r,z} \right)\frac{{{\hat{\mathbf x}} + m{\hat{\mathbf y}}}}{{\sqrt {1 + \left| m \right|^2} }}{\mathrm{exp}}\left( {ikz + i\ell \theta } \right)$$

**Fig. 3:**
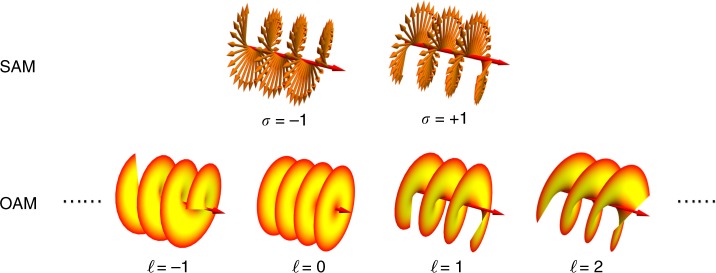
The OAM of light beams is revealed by the phase distributions and the SAM by the polarizations. The phase distributions for various eigenstates of OAM and the polarizations (right- and left-handed circularly polarized) for the two eigenstates of SAM are illustrated

The average SAM and OAM can be derived as^69,70^

5 $$\frac{{\mathbf{S}}}{W} = \frac{\sigma }{\omega }\frac{{\mathbf{k}}}{k},\frac{{\mathbf{L}}}{W} = \frac{\ell }{\omega }\frac{{\mathbf{k}}}{k}$$

where the power density $W = \frac{{g\omega }}{2}\left( {\tilde \varepsilon \left| {\mathbf{E}} \right|^2 + \tilde \mu \left| {\mathbf{H}} \right|^2} \right)$ and $\sigma = \frac{2{{\rm Im}({m})}}{{1 + \left| m \right|^2}}$. *σ* = +1 (−1) and 0 correspond to left (right) circularly polarized light and linearly polarized light, respectively. Thus, Eq. (4) reveals that left (right) circularly polarized light carries an SAM of + ℏ (−ℏ) per photon; the light with Hilbert factor exp(i*ℓθ*) carries an OAM of *ℓ*ℏ (*ℓ* = 0, ±1, ±2, …) per photon, where “ ± ” reveals the chirality of the vortex, as demonstrated in Fig. 3. This is consistent with the AM quantization in quantum optics, i.e., the eigenvalues of SAM and OAM for the photon eigenstate yield $\hat L_z\left| \psi \right\rangle = \ell \hbar \left| \psi \right\rangle$ and $\hat S_z\left| \psi \right\rangle = \sigma \hbar \left| \psi \right\rangle$. Therefore, the phase factor exp(i*ℓφ*) provides a basic frame of VBs.

### Polarization and vector vortices

**Fig. 4:**
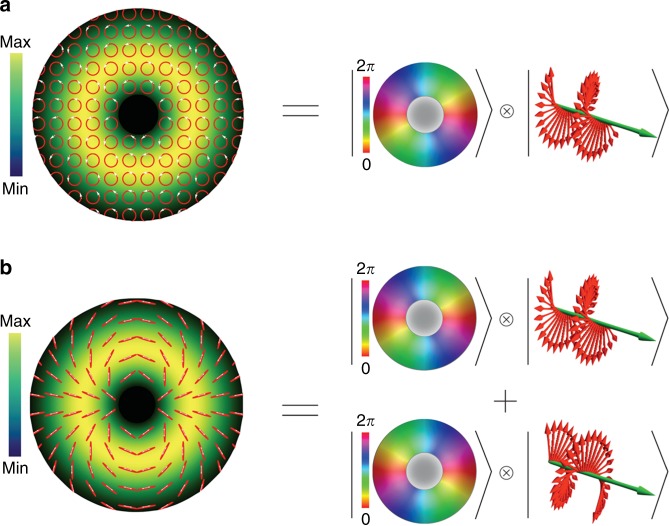
Formation of vector beam with space-polarization nonseparability. **a** Circularly polarized OV with an azimuthally varying phase distribution. Such a state is considered separable, as it can be represented as the product of a spatially varying vortex phase and a polarization state vector. **b** Spider-like vector vortex represented as the superposition of the state of **a** with another state with the opposite phase variation and the opposite circular polarization. From ref. ^71^. Reprinted with permission from AAAS

The previous part focuses on the scalar light field, where the polarization is separable from the space. In scalar vortices, there are topological spatial phase structures, but the polarization is unchanged; e.g., Fig. 4a shows that a circularly polarized OV can be expressed as the product of a spatially varying vortex phase state and a circular polarization state^71^. If the polarization state has a spatially varying vector distribution forming vortex-like patterns, then the corresponding optical field is called polarization vortices or vector vortices, and the corresponding singularity is called a polarization singularity or a vector singularity^72,73^. Based on the various topological disclinations of polarization, vector vortices can be categorized into many types, such as C-point, V-point, lemons, star, spider, and web, according to the actual vector morphology^74^. In contrast to the phase vortices carrying OAM, the vector vortices are always related to a complex SAM-OAM coupling; e.g., Fig. 4b shows a spider-like vector OV formed by the superposition of opposite phase variations and opposite circular polarizations, where the total OAM is zero due to the sum of the two opposite phase variations but there is a complex SAM entangled with the space^71^.

### Classical models of OVs

#### LG and Hermite–Laguerre–Gaussian modes

**Fig. 5:**
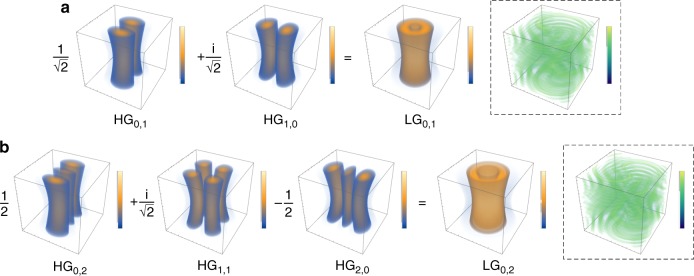
Decomposition of LG vortex beams. Examples of the decomposition of LG modes (LG_0,1_ (**a**) and LG_0,2_ (**b**)) into HG modes according to Eq. (7), where the insets in the dotted box show the corresponding vortex phase distributions

LG modes with circular symmetry are the earliest reported VBs carrying OAM^16^ and can be included in the general family of Hermite–Laguerre–Gaussian (HLG) modes with elliptical vortices^75–77^, thus accommodating the transform from the HG to LG mode, which has recently played increasingly important roles because the exploration of the more general structure of OVs always leads to novel applications:

6 $$\begin{array}{c}{\mathrm{HLG}}_{n,m}\left( {\left. {{\mathbf{r}},z} \right|\alpha } \right) = \frac{1}{{\sqrt {2^{N - 1}n!m!} }}\exp \left( { - \pi \frac{{\left| {\mathbf{r}} \right|^2}}{w}} \right){\mathrm{HL}}_{n,m}\left( {\left. {\frac{{\mathbf{r}}}{{\sqrt \pi w}}} \right|\alpha } \right)\\ \times \exp \left[ {{\mathrm{i}}kz + {\mathrm{i}}k\frac{{r^2}}{{2R}} - {\mathrm{i}}\left( {m + n + 1} \right)\vartheta } \right]\end{array}$$

where HL_*n*,*m*_(·) is a Hermite–Laguerre (HL) polynomial^75^, ${r=(x,y)^{\mathrm{T}}} = {(r\,\cos{\varphi}, r\,\sin {\varphi})^{\mathrm{T}}}$, $R(z) = (z_R^2 + z^2)/z$, $kw^2(z) = 2(z_R^2 + z^2)/z_R$, $\vartheta (z) = \arctan (z/z_R)$, and *z*_*R*_ is the Rayleigh range. For *α* *=* 0 or *π*∕2, the HLG_*n*,*m*_ mode is reduced to the HG_*n*,*m*_ or HG_*m*,*n*_ mode. For *α* *=* *π*∕4 or 3*π*∕4, the HLG_*n,m*_ mode is reduced to LG_*p*,±*ℓ*_ mode [$p = \min \left( {m,n} \right)$, $\ell = m - n$]. For the other interposed states, the HLG mode has multiple singularities with a total TC of *ℓ*. As illustrated in Fig. 5, the LG_*p*,*ℓ*_ mode can be decomposed into a set of Hermite–Gaussian (HG) modes^16,17^:

7 $${\mathrm{LG}}_{p, \pm \ell }\left( {x,y,z} \right) = \mathop {\sum}\limits_{K = 0}^{m + n} {({\pm{\mathrm{i}}})^Kb\left( {n,m,K} \right) \cdot } {\mathrm{HG}}_{m + n - K,K}\left( {x,y,z} \right)$$

8 $$b\left( {n,m,K} \right) = \left[ {\frac{{\left( {N - K} \right)!K!}}{{2^Nn!m!}}} \right]^{1/2}\frac{1}{{K!}}\left. {\frac{{{\mathrm{d}}^K}}{{{\mathrm{d}}t^K}}\left[ {\left( {1 - t} \right)^n\left( {1 + t} \right)^m} \right]} \right|_{t = 0}$$

which also interprets the transformation to an LG_*p*,*ℓ*_ mode from an HG_*n*,*m*_ mode through an astigmatic mode converter (AMC)^17^.

#### Helical-Ince–Gaussian and singularities hybrid evolution nature mode

The Ince-Gaussian (IG) mode^78^ is the eigenmode of the paraxial wave equation (PWE) separable in elliptical coordinates (*ξ*, *η*)^79^:

9 $$\begin{array}{l}{\mathrm{IG}}_{u,v}^{e,o}\left( {\left. {x,y,z} \right|\epsilon } \right) = \\ \frac{{C^{e,o}}}{w}I_{u,v}^{e,o}\left( {{\mathrm{i}}\xi ,\epsilon } \right)I_{u,v}^{e,o}\left( {\eta ,\epsilon } \right)\exp \left( { - \frac{{x^{2}\ +\ y^{2}}}{{w^2}}} \right)\exp \left[ {{\mathrm{i}}kz + {\mathrm{i}}k\frac{{x^{2}\ +\ y^2}}{{2R}} - {\mathrm{i}}\left( {u + 1} \right)\vartheta } \right]\end{array}$$

where *C*^*e,o*^ are normalization constants (the superscripts *e* and *o* refer to even and odd modes), $I_{u,v}^{e,o}\left( { \cdot ,\epsilon } \right)$ are the even and odd Ince polynomials, with $0\ <\ v\ <\ u$ for even functions, $0\ <\ u\ <\ v$ for odd functions, and $\left( { - 1} \right)^{u - v} = 1$ for both, and $\epsilon\in \left( {\left. {0,\infty } \right)} \right.$ is the eccentricity. The special superposition of these modes can form a multi-singularity array with OAM, named the helical-IG (HIG) modes^80–82^:

10 $${\mathrm{HIG}}_{u,v}^ \pm \left( {\left. {x,y,z} \right|\epsilon } \right) = {\mathrm{IG}}_{u,v}^e\left( {\left. {x,y,z} \right|\epsilon } \right) \pm {\mathrm{i}} \cdot {\mathrm{IG}}_{u,v}^o\left( {\left. {x,y,z} \right|\epsilon } \right)$$

which carries multiple singularities with unit TC, having a total TC of *v*. Sharing the singularities hybrid evolution nature (SHEN) of the HIG and HLG modes, the SHEN mode is a very general family of structured Gaussian modes including the HG, LG, HLG, and HIG modes, the expression of which is^83^

11 $$\begin{array}{c}{\mathrm{SHEN}}_{n,m}\left( {x,y,z|\beta ,\gamma } \right) = \mathop {\sum}\limits_{K = 0}^N {{\mathrm{e}}^{{\mathrm{i}}\beta K}b\left( {n,m,K} \right)} \\ \cdot \left\{ {\begin{array}{*{20}{c}} {\left( { - {\mathrm{i}}} \right)^K{\mathrm{IG}}_{N,N - K}^e\left( {x,y,z|\epsilon = 2{\mathrm{/}}\tan ^2\gamma } \right),{\mathrm{for}}\left( { - 1} \right)^K = 1} \\ {\left( { - {\mathrm{i}}} \right)^K{\mathrm{IG}}_{N,N - K + 1}^o\left( {x,y,z|\epsilon = 2{\mathrm{/}}\tan ^2\gamma } \right),{\mathrm{for}}\left( { - 1} \right)^K \ne\;1} \end{array}} \right.\end{array}$$

The SHEN mode is reduced to the HIG mode when *β* = ±*π*/2, to the HLG mode when *γ* = 0, to the HG mode when (*β*, *γ*) = (0,0) or (*π*, 0), and to the LG mode when (*β*, *γ*) = (±*π*/2, 0). In addition, there is a graphical representation, the so-called SHEN sphere, to visualize the topological evolution of multi-singularity beams. Thus, the SHEN mode has great potential to characterize more general structure beams.

#### Bessel and Mathieu modes

Using the non-diffraction assumption in solving the PWE, we can also solve a set of eigenmodes. Under separable conditions in circular coordinates, the Bessel mode can be obtained as^84^

12 $${\mathrm{B}}_\ell \left( {r,\theta ,z} \right) = J_\ell \left( {\mu r} \right)\exp \left( {{\mathrm{i}}\ell \theta } \right)\exp \left( {{\mathrm{i}}kz} \right)$$

Bessel beams with *ℓ* ≠ 0 are VBs carrying *ℓ*ℏ OAM. Another non-diffraction solution separable in elliptical coordinates is the Mathieu modes^85^,

13 $${\mathrm{M}}_m^e\left( {\left. {x,y,z} \right|\epsilon } \right) = C_{m}{\mathrm{Je}}_{m}\left( {\xi ,\epsilon } \right){\mathrm{ce}}_{m}\left( {\eta ,\epsilon } \right)\exp \left( {{\mathrm{i}}k_zz} \right)$$

14 $${\mathrm{M}}_{m}^{o}\left( {\left. {x,y,z} \right|\epsilon } \right) = S_{m}{\mathrm{Jo}}_{m}\left( {\xi ,\epsilon } \right){\mathrm{se}}_{m}\left( {\eta ,\epsilon } \right)\exp \left( {{\mathrm{i}}k_zz} \right)$$

where *C*_*m*_ and *S*_*m*_ are normalization constants, Je_*m*_ and Jo_*m*_ are radial Mathieu functions, and ce_*m*_ and se_*m*_ are angular Mathieu functions. Analogous to deriving the HIG mode, a helical Mathieu (HM) beam^86^ can carry multiple singularities and complex OAM^87^.

15 $${\mathrm{HM}}_m^ \pm \left( {\left. {x,y,z} \right|\epsilon } \right) = {\mathrm{M}}_m^e\left( {\left. {x,y,z} \right|\epsilon} \right) \pm {\mathrm{i}} \cdot {\mathrm{M}}_m^o\left( {\left. {x,y,z} \right|\epsilon} \right)$$

High-order Bessel and HM beams are often called non-diffractive VBs, whose unique properties have been extended to a great number of applications, such as particle assembly and optical communication^88,89^.

#### SU(2) geometric modes

**Fig. 6:**
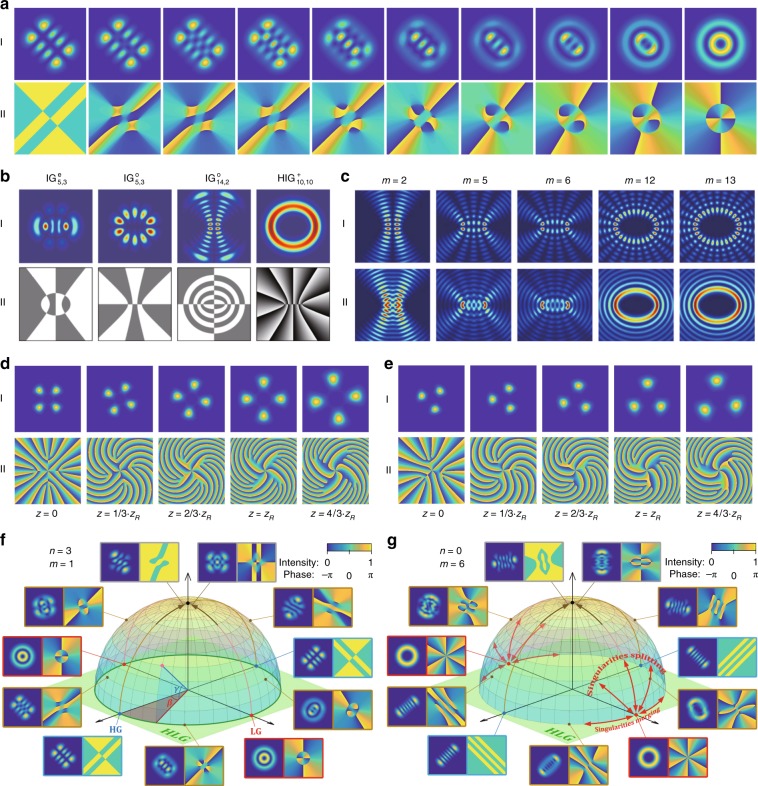
Classical models of paraxial VBs. **a** Evolution of the (I) intensity and (II) phase distributions of HLG modes as interposed states between HG and LG modes; **b** various (I) intensity and (II) phase distributions of IG and HLG modes^82^; **c** intensity distributions of a selection of (I) odd and (II) helical Mathieu beams^88^. (I) Intensity and (II) phase distributions of SU(2) vortex geometric modes for Ω = 1/4 (**d**) and Ω = 1/3^97^ (**e**). SHEN spheres with orders of (*n*, *m*) = (3, 1) (**f**) and (*n*, *m*) = (0, 6) (**g**) along with represented mode (phase) fields at selected points^83^. **b** Reproduced from ref. ^82^, with the permission of AIP Publishing. **c** Reprinted with permission from ref. ^88^, OSA Publishing. **d**, **e** Reprinted with permission from ref. ^97^, OSA Publishing. **f**, **g** Reprinted with permission from ref. ^83^, OSA Publishing

When a resonator cavity fulfils the reentrant condition of a coupled quantum harmonic oscillator in SU(2) Lie algebra^90^, the laser mode undergoes frequency degeneracy with a photon performing as an SU(2) quantum coherent state coupled with a classical periodic trajectory^91^, which is called an SU(2) geometric mode (GM)^92^. The frequency degeneracy means that ${\mathrm{\Delta }}f_{\mathrm{T}}/{\mathrm{\Delta }}f_{\mathrm{L}} = P/Q = {\mathrm{\Omega }}$ should be a simple rational number, where *P* and *Q* are two coprime integers, and ${\mathrm{\Delta }}f_{\mathrm{T}}$(${\mathrm{\Delta }}f_{\mathrm{L}}$) is the longitudinal (transverse) mode spacing. The wave-packet function of a planar GM is given by^92^

16 $$\Psi _{n_0}^M\left( {x,y,z;\phi _0\left| \Omega \right.} \right) = \frac{1}{{2^{M/2}}}\mathop {\sum}\limits_{K = 0}^M {\sqrt {\frac{{M!}}{{K!\left( {M - K} \right)!}}} } \cdot {\mathrm{e}}^{{\mathrm{i}}K\phi _0} \cdot \psi _{n_0 + Q \cdot K,0,s_0 - P \cdot K}^{\left( {{\mathrm{HG}}} \right)}\left( {x,y,z} \right)$$

where phase *ϕ*_0_ is related to the classical periodic trajectory. $\psi _{n,m,s}^{\left( {{\mathrm{HG}}} \right)}$ represents the HG_*n*,*m*_ mode considering the frequency-dependent wavenumber $k_{n,m,s} = 2\pi f_{n,m,s}/c$, where $f_{n,m,s} = s \cdot \Delta f_{\mathrm{L}} + \left( {n + m + 1} \right) \cdot \Delta f_{\mathrm{T}}$. If the HG bases are transformed into LG bases, then the circular GM is obtained^92^:

17 $$\Phi _{n_0}^M\left( {x,y,z;\phi _0\left| \Omega \right.} \right) = \frac{1}{{2^{M/2}}}\mathop {\sum}\limits_{K = 0}^M {\sqrt {\frac{{M!}}{{K!\left( {M - K} \right)!}}} } \cdot {\mathrm{e}}^{{\mathrm{i}}K\phi _0} \cdot \psi _{0, \pm \left( {n_0 + Q \cdot K} \right),s_0 - P \cdot K}^{\left( {{\mathrm{LG}}} \right)}\left( {x,y,z} \right)$$

where $\psi _{p,\ell ,s}^{\left( {{\mathrm{LG}}} \right)}$ represents the LG_*p*,*ℓ*_ mode considering the frequency-dependent wavenumber. The vortex circular GM has many unique properties, such as an exotic 3D structure, multiple singularities, and fractional OAM^92,93^. Note that there are other types of SU(2) modes related to OAM with special properties, such as Lissajous modes^94^, trochoidal modes^95^, polygonal VBs^96^, and SU(2) diffraction lattices^97^ as shown in Fig. 6d, e.

The above forms are classical VBs in free space, which are just optical modes carrying OAM. In addition, there are OVs that are formed by non-OAM beams, as reviewed in the following.

#### Optical Möbius strips

A direct idea is to arrange the optical parameter into the form of Möbius strips, one of the classical topological models. This type of OV is called an optical Möbius strip (OMS). A simple vortex phase with integer TC can be seen as a phase OMS. In addition to phase vortices, more OMSs can be obtained by arranging the polarization: the major and minor axes of the polarization ellipses that surround singular lines of circular polarization in three-dimensional optical ellipse fields can be organized into an OMS, as theoretically proposed^98,99^ and experimentally observed^49^. Currently, multitwist OMSs can be controlled in both paraxial and nonparaxial vector beams^56,100^. By combining other spatial and optical parameters into OMSs, more complex structures, such as 3D solitons and topological knots, can be proposed for OVs^101^.

#### Vortex knots

**Fig. 7:**
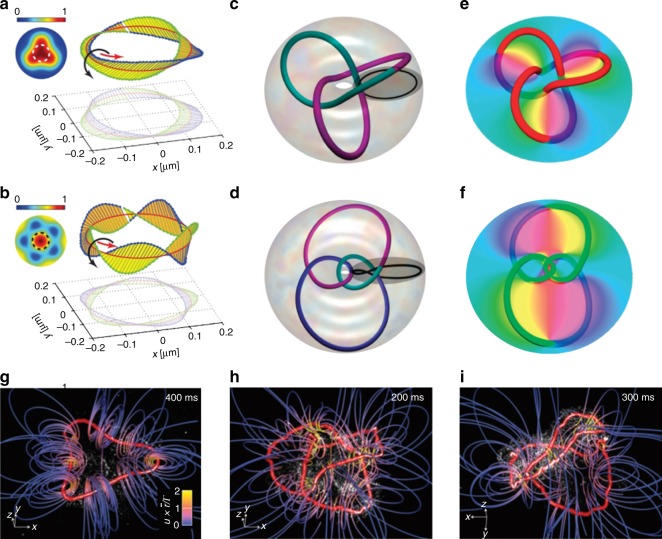
Classical models of spatial nonparaxial OVs. Polarization topology of optical Möbius strips with twisted TCs of –1/2 and –3/2 (**a**, **b**)^56^. Nodal trefoil knot and pigtail braid knot OVs (**c**, **d**) and corresponding phase distributions (**e**, **f**)^104^. Optical vortex knots of a threefold distorted loop (**g**), a trefoil knot (**h**), and a pair of linked rings (**i**)^103^. **a**, **b** From ref. ^56^. Reprinted with permission from AAAS. **c**–**f** Reprinted by permission from Nature Physics^104^, Copyright (2019). **g**–**i** Reprinted by permission from Nature Physics^103^, Copyright (2019)

The vortex core of an OV can not only be distributed along the propagation axis of a beam but also form closed loops, links and knots embedded in a light field^102^. As a new form of OVs, vortex knots have stimulated many experimental observation and theoretical studies on the dynamics of knotted vortices^102,103^. Vortex knots can also show many homologies, such as pigtail braid and Nodal trefoil knots^104^ as shown in Fig. 7c–f. Currently, researchers have realized the isolated manipulation and temporal control of optical vortex knots^104,105^.

There are many other forms of OVs that cannot be fully covered in this paper. For instance, there are many free-space VB modes that carry OVs and OAM, such as elegant HLG beams^106^, Airy beams^107^, Pearcey beams^108^, and parabolic beams^109^. There are many morphologies of the non-beam spatial distribution of OVs with singularities fractality^110^. It is highly expected that many new formations of OVs will be reported and investigated in future explorations.

### Properties of VBs

#### Reflection and refraction

The reflection of a VB generally does not satisfy the classical reflection law, i.e., the angle of incidence *θ*_i_ does not equal the angle of reflection *θ*_r_. Instead, the reflected light has a spatial deflection effect related to the OAM of the VB^111^. The difference between *θ*_i_ and *θ*_r_ is related to the OAM of the beam, obeying the generalized law of reflection^41^

18 $$\sin \left( {\theta _{\mathrm{r}}} \right) - \sin \left( {\theta _{\mathrm{i}}} \right) = \frac{\lambda }{{2\pi n}}\frac{{{\mathrm{d}}\phi }}{{{\mathrm{d}}x}}$$

where *λ* and *ϕ* are the wavelength and phase of the light beam, respectively, and *n* is the refractive index of the medium. In addition, the refraction of VBs does not satisfy Snell’s law, i.e., *n*_t_sin*θ*_t_ ≠ n_i_sin*θ*_i_. The refraction is related not only to the angles of incidence and refraction (*θ*_i_ and *θ*_t_) and the refractive indices but also to the OAM, obeying the generalized law of refraction^41^

19 $$\sin \left( {\theta _{\mathrm{t}}} \right)n_{\mathrm{t}} - \sin \left( {\theta _{\mathrm{i}}} \right)n_{\mathrm{i}} = \frac{\lambda }{{2\pi }}\frac{{{\mathrm{d}}\phi }}{{{\mathrm{d}}x}}$$

#### Interference

For conventional laser beams, the equal-inclination interference pattern is equispaced fringes, and the equal-thickness interference pattern is Newton’s rings. However, for a VB, the pattern of equal-inclination interference with a plane wave is not equispaced fringes but fringes with bifurcation at the singularity of the vortex, and the morphology of the bifurcation is related to the OAM of the beam^66^. The equal-thickness interference pattern of a VB with a plane wave is not Newton’s rings but spiral stripes extending outward from the vortex singularity, the number of which is related to the OAM^112^. The self-interference pattern can also show some bifurcation fringes^112^. These special interference fringes can be used in detection and measurement methods of vortices.

#### Diffraction

VBs have unique diffraction properties, the aperture diffraction patterns of which are coupled with the actual OAM. Since Hickmann et al.^40^ unveiled in 2010 the exotic lattice pattern in triangular-aperture far-field diffraction of VBs, it has been used as an effective method for OAM detection and measurement of femtosecond vortices^113^, non-integer charge vortices^114^, and elliptical VBs^115^. Many other unique far-field diffraction patterns were investigated through a slit^116^, a square aperture^117^, a diamond-shaped aperture^118^, a circular aperture^119^, an off-axis circular aperture^120^, an isosceles right triangular aperture^121^, a sectorial screen^122^, and so on. The Fresnel diffraction of VBs was also studied^123^. Some special VBs, such as vector VBs^124^ and SU(2) VBs^97^, can even bring about special lattice structures in diffraction patterns. These special diffraction patterns can be used in vortex detection and measurement methods.

#### Polarization

**Fig. 8:**
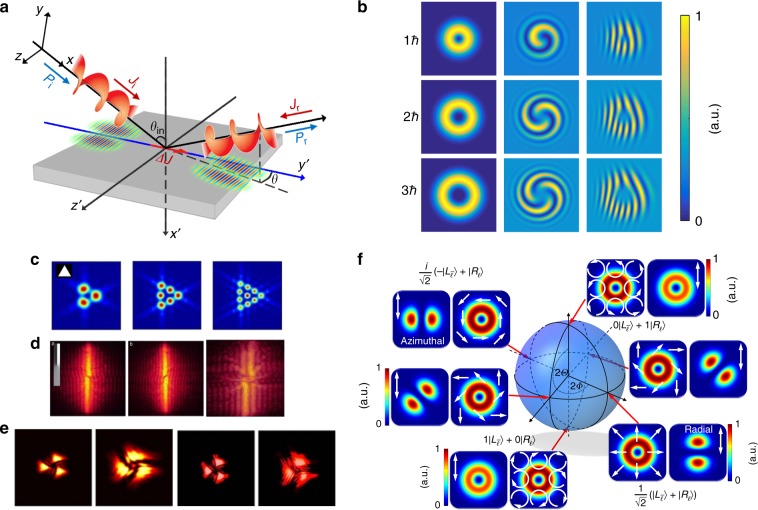
Reflection, interference, diffraction, and polarization of VBs. **a** Abnormal reflection of a VB^111^. **b** LG VBs with different TCs (first column) and corresponding interference patterns with a co-axis coherent planar wave (second column) and an inclined coherent planar wave (third column). Far-field diffraction patterns of VBs through a triangular aperture^40^ (**c**) and a single slit^116^ (**d**). **e** Near-field diffraction pattern of a VB^123^. **f** Polarization distribution of vector VBs on the HPS^127^. **a** Reprinted with permission from ref. ^111^. Copyright (2019) by the American Physical Society. **c** Reprinted with permission from ref. ^40^. Copyright (2019) by the American Physical Society. **d** Reprinted with permission from ref. ^116^, Copyright (2019), with permission from Elsevier. **f** Reprinted by permission from Nature Photonics^127^, Copyright (2019)

The polarization states of conventional beams can be represented on the Poincaré sphere. VBs can have complex transverse structures involving polarization vortices. Upon combining structured polarization with VBs, the vector VBs can demonstrate more amazing properties and more extensive applications^74^. To characterize a classical family of vector VBs, Holleczek et al. proposed a classical-quantum-connection model to represent cylindrically polarized beams on the Poincaré sphere^125^; this model was then extended to the high-order Poincaré sphere (HPS)^126^, which can reveal SAM-OAM conversion and more exotic vector beams, including radial and azimuthal polarization beams. In an experiment, controlled generation of HPS beams was realized^127^ as illustrated in Fig. 8f. As an improved formation of the HPS, the hybrid-order Poincaré sphere was theoretically proposed^128^, and the corresponding experimental controlled generation methods were also presented^129,130^.

#### Quantum properties

Twisted photons^31^ are associated with the quantum behaviour of macroscopic VBs. Akin to the conventional Heisenberg uncertainty, there is also the formation of uncertainty for twisted photons; i.e., the product of the uncertainties in the angle and the OAM is bounded by Planck’s constant, Δ*ϕ*Δ*L* ≥ ℏ/2^131,132^. The general Fourier relationship between the angle and the OAM of twisted photons was also investigated^133^. In contrast to the polarization-entangled state with two dimensions, the OAM-entangled state can be high dimensional as $\left| \Psi \right\rangle {\mathrm{ = }}\mathop {\sum}\limits_\ell {c_\ell \left| \ell \right\rangle _{\mathrm{A}}\left| { - \ell } \right\rangle _{\mathrm{B}}}$^134^. Combining the polarization and OAM of the photon, more complex SAM–OAM entangled photon pairs were realized^47,135^. There are many other new quantum properties related to OAM beams, such as the spin–orbit interaction^136–138^, the Hanbury–Brown–Twiss effect^139^, quantum interference^140,141^, and the spin Hall effect^142,143^.

### Measurements of OVs

As mentioned above, OVs can be measured by adopting the interference and diffraction properties of VBs. Counting the stripes and lattices in the special interferogram and diffraction patterns serves as a toolkit to measure the TC, OAM, and singularity distributions of corresponding OVs. In addition, for measuring phase vortices, one can use a spatial light modulator (SLM) to carry out phase transformations, reconstructing the target phase to detect the TC and OAM. Typical realizations include the forked diffraction grating detector^144^, the OAM sorter^145^, and spiral transformation^146^. For polarization vortices, the measurement should also consider the detection of the vector field. By introducing a space-variant structure into a half-wave plate to modulate the polarization, the TC of the polarization singularity in vector VBs can be measured^147^. For measuring more properties of vector OVs, Forbes’ group introduced quantum measurement methods to classical light and realized more precise measurement of properties such as the non-separability, SAM–OAM coupling, and vector factors of vector beams^148,149^, which is widely applicable to more structured OVs.

## Progress in vortex generation, tuning, and manipulation

### Brief review of vortex generation

The vortex generation methods can be divided into passive vortex generators (converting the fundamental Gaussian beams into VBs by using dynamic or geometric phase plates, metasurfaces, SLMs, etc.) and active vortex laser generators (such as free space or fibre vortex lasers and nanointegrated OAM generators)^61,112,150^. There have already been some recent reviews on vortex and OAM beam generation^61–63,112,150^. However, a review focused on vortex generation with tunable and multi-singularity properties is rare. Hereinafter, we specifically review active vortex generation with tunable properties, including wavelength-, temporal-, and OAM-tunable beams. In particular, the OAM-tunable beams include TC-tunable and multi-singularity-tunable beams.

### Wavelength- and OAM-tunable VBs

OAM-tuning of VBs can be realized by gain-loss control^151^, off-axis pumping^92,152^, or the use of a spiral phase plate (SPP)^153^, a Q-plate^154,155^, or an SLM^144^. A wavelength-tunable VB can be achieved by designing special liquid crystal devices^156^, microcavities^157^, or on-chip gratings^158^ or using nonlinear frequency conversion^159,160^. However, more methods to simultaneously realize wavelength and OAM tuning for novel applications, such as high-capacity optical communication using wavelength- and mode-division multiplexing, are still required.

**Fig. 9:**
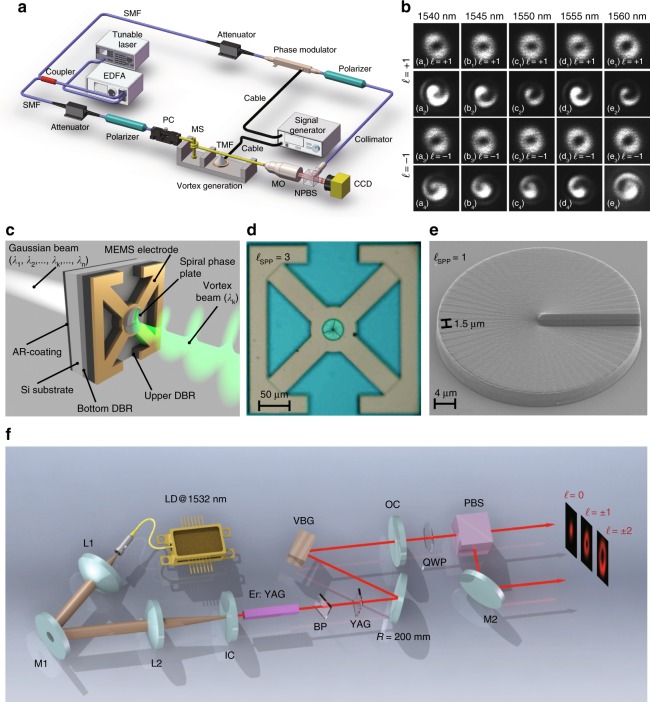
Generation of wavelength- and OAM-tunable CW VBs (I). Generation methods of wavelength- and OAM-tunable VBs using an acousto-optic fibre grating (**a**), along with the experimental results^161^ (**b**), an SPP-embedded MEMS filter system^162^ (**c**–**e**), and a VBG in a hollow-pumped solid-state laser^163^ (**f**). Reprinted with permission from refs. ^161–163^, OSA Publishing

**Fig. 10:**
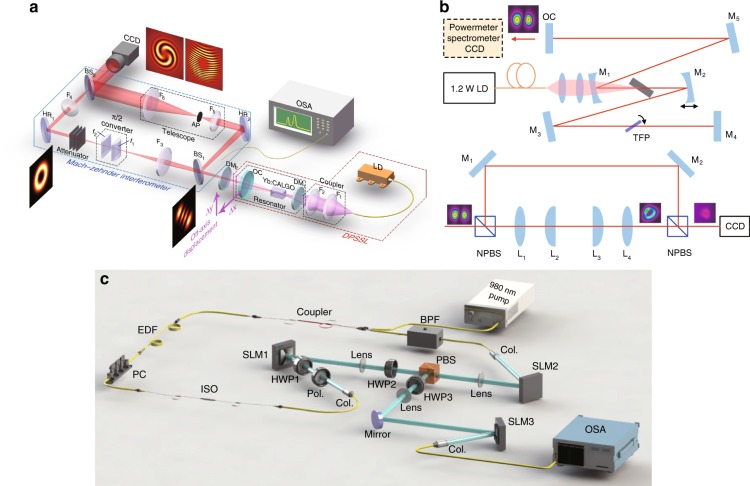
Generation of wavelength- and OAM-tunable CW VBs (II). Generation methods of wavelength- and OAM-tunable VBs using a dual-off-axis pumped Yb:CALGO laser system^165,166^ (**a**), intracavity birefringent plate rotation^167^ (**b**), and a “Gauss-OAM-Gauss” beam conversion system in a fibre laser^168^ (**c**). **a** From refs. ^165,166^. Reproduced by permission of IOP Publishing. **b** Reprinted with permission from ref. ^167^, OSA Publishing

In 2016, Zhang’s group^161^ presented a wavelength- and OAM-tunable system by employing a tunable fibre laser with an acousto-optic fibre grating with a wavelength-tunable range of 1540–1560 nm and an OAM of ± 1ℏ, as shown in Fig. 9a, b. In 2017, Lyubopytov et al.^162^ designed a micro-electro-mechanical (MEMS) filter system realizing vortex generation with a wavelength-tunable width of 37.5 nm and an OAM of 0~3ℏ. In the same year, Liu et al.^163^ reported a ring-pumped Er:YAG solid-state laser generating an 8.4-nm wavelength-tunable width and 0~±2ℏ OAM-tunable VB. In 2018, Yao et al.^164^ invented a new optical fibre combiner for combining two polarization-controllable fundamental modes into a VB with chiral control, obtaining a 30-nm wavelength-tunable width and 0~±1ℏ OAM. Our group^165^ proposed solid-state vortex generation utilizing a dual-off-axis pumped ultra-wide-band Yb:CALGO laser, reaching a wavelength-tunable width of over 10 nm and an OAM range of 0~±15ℏ, as depicted in Fig. 10a. This system was adapted to generate tunable dual-wavelength VBs^166^. Recently, Wang et al.^167^ improved the output efficiency and reduced the threshold of a similar system by using a Z-cavity and a birefringent plate in the cavity design, and a 14.5-nm wavelength-tunable width and a 0~±14ℏ OAM range were achieved. Wang’s group^168^ designed and implemented a fibre-space coupling vortex laser system, where a wavelength-tunable range of 1530–1565 nm and an OAM of 0~±10ℏ were achieved.

In addition to the abovementioned wavelength- and OAM-tunable OVs from lasers, there are vortex generators that are known to be intrinsically broadband, which can also be used to obtain wavelength- and OAM-tunable OVs. For instance, vortex generation from anisotropic solid crystals, both uniaxial and biaxial, can be related to complex SAM–OAM coupling and gain competition effects, leading to the tunability of vector OVs^72,73,169^. Similar tunable OVs can be generated in chiral liquid crystals in the regime of circular Bragg reflection^170,171^. Taking advantage of space-variant anisotropic liquid crystals that can be electrically controlled, wavelength- and OAM-tunable OVs can be generated within a wide spectral tunable range^156,172^. Overall, a wider tunable range controlled by a more convenient OV generation method is still required in the current explorations.

### Pulsed VBs

High-peak-power pulsed VBs with different levels of duration have great potential for use in advanced applications, such as optical machining^173,174^, nonlinear optics^25,26,48,49^, strong-field physics^175^, and optical tweezers^176^. Hereinafter, we review the recent compact and effective pulsed VB sources.

#### Nanosecond-level VBs

The generation of nanosecond-level VBs has always been combined with Q-switched lasers. An earlier method involved selecting the LG modes using an intracavity aperture in a Q-switched solid-state laser^177^, with which obtaining high mode purity is difficult. In 2013, Kim et al.^178^ used an etalon and a Brewster plate in an acoustic-optic Q-switched laser and generated high-quality chirality-controlled LG beams with an ~250 μJ pulse energy and an ~33 ns duration. In 2016, Zhao et al.^179^ controlled the pump position in an Er:YAG acoustic-optic Q-switched laser, generating a nearly 1-mJ and 50-ns pulsed VB. In 2017, Chen’s group^180^ designed a nanosecond vortex laser system employing a compact Nd:YVO_4_/Cr^4+^:YAG passively Q-switched laser with an external AMC. In 2018, He et al.^181^ presented a Cr,Nd:YAG self-Q-switched microchip laser to directly generate low-threshold nanosecond high-repetition-rate vortex pulses without an AMC, where the chirality was controlled by a tilted output mirror. Our group^182^ recently reported a pulsed vortex output directly from a room-temperature diode-pumped Er,Yb:glass microchip laser with an extremely compact structure.

#### Picosecond-level VBs

Picosecond-level VBs have always been realized in a mode-locking laser using a narrow-band gain medium. In 2011, Koyama et al.^183^ realized a VB in a stressed Yb-doped fibre amplifier seeded by a picosecond mode-locked Nd:YVO_4_ laser with a pulse width of 8.2 ps and a peak power of 34.2 kW. However, the master oscillator power-amplifier structure limited the compactness. The discovery of a self-mode-locking effect in neodymium-doped crystals provided an alternative way to generate picosecond pulses with a quite compact structure^184^. In 2009, Liang et al.^185^ reported an OV with a pulse width of 20–25 ps and a repetition rate of 3.5 GHz using off-axis-pumped self-mode-locked Nd:GdVO_4_ lasers with an AMC. In 2013, the same group^186^ improved this system via cavity control and realized the self-mode-locked SU(2) vortex GM with pulse widths of 22.2 ps and 21.1 ps for Ω = 1/4 and Ω = 1/3, respectively^92^. In 2017, Tung et al.^187^ reported the direct generation of picosecond large-OAM SU(2) vortex pulses in self-mode-locking Nd:YVO_4_ lasers without the help of an AMC, which largely enhanced the compactness. In 2018, Huang et al.^188^ reported an 8.5 ps pulsed VB generated from a mode-locked fibre laser, where the polarization could be controlled at arbitrary states on the HPS.

#### Femtosecond-level VBs

**Fig. 11:**
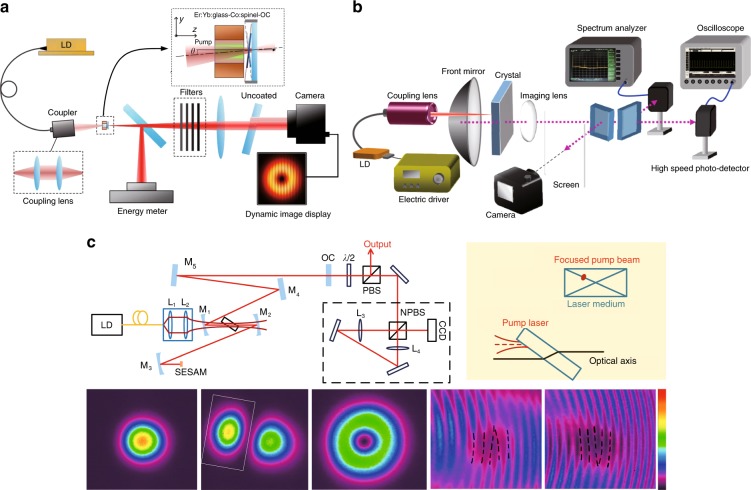
Generation of wavelength- and OAM-tunable pulsed VBs. Generation methods of a room-temperature diode-pumped Er,Yb:glass microchip nanosecond laser^182^ (**a**), picosecond-level VBs in a self-mode-locked Nd:YVO_4_ laser^187^ (**b**), and femtosecond VBs in an SESAM mode-locking laser^193^ (**c**). **a** ©(2019) IEEE. Reprinted, with permission, from ref. ^182^. **b** Reprinted with permission from ref. ^187^, OSA Publishing. **c** From ref. ^193^. Reproduced by permission of IOP Publishing

In contrast to picosecond pulses, femtosecond pulse generation always requires more extreme operating conditions, such as a tightly focused pumping spot, a wide emission band, and a high nonlinear coefficient of the gain medium. Utilizing the external modulation method, flexible temporal shaping of femtosecond VBs was recently realized^189^. Considering the improvements in compactness and cost, the self-mode-locking laser oscillator scheme is still desirable. In 2013, Chen’s group^190^ reported a self-mode-locked monolithic Yb:KGW laser with a duration of 850 fs and a repetition rate of 22.4 GHz. In 2016, they^191^ improved the system to directly generate a sub-picosecond VB carrying OAM by selective pumping. In 2018, Zhang et al.^192^ proposed an all-fibre mode-locked femtosecond LG_0,±1_ vortex laser with a pulse width of 398 fs. In the same year, Wang et al.^193^ realized direct emission of an ultrafast LG_0,±1_ VB via a z-type cavity design in an SESAM mode-locking Yb:QX laser with a pulse width of 360 fs, as shown in Fig. 11c. These structures have recently been improved by using a Yb:KYW oscillator with a defect-spot mirror, obtaining a 298-fs VB^194^. Direct generation of sub-100-fs VBs may be a future target.

### Complex OAM manipulation

In addition to TC-tunable VBs, beams with multiple singularities can induce exotic tunable OAM. The multi-singularity optical field with a vortex array is also known as a vortex lattice or a vortex crystal^5,29,73^. Strong requirements of multi-singularity beams have been put forward because of the boom of special applications such as multiple particle manipulation^82,195^, 3D displays^196^, and optical modulation and communication^197^.

**Fig. 12:**
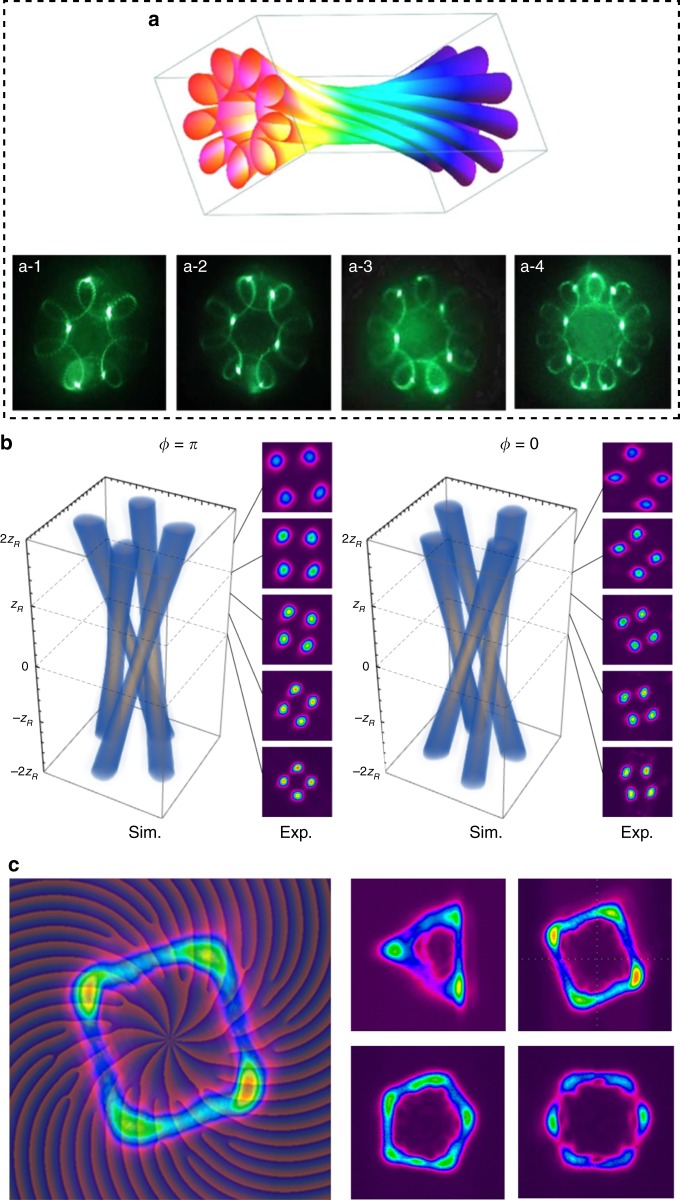
Exotic SU(2) structured multi-singularity VBs. Theoretical and experimental investigations of special multi-singularity VBs: **a** trochoidal vortex modes^95^, **b** the vortex SU(2) GM^204^, and **c** polygonal VBs^96^. **a** Reprinted with permission from ref. ^95^. Copyright (2019) by the American Physical Society. **b** Reprinted with permission from ref. ^204^, OSA Publishing. **c** © (2019) IEEE. Reprinted, with permission, from ref. ^96^

A singularity splitting phenomenon was found in an AMC when the phase matching condition in the AMC was not satisfied^198,199^. A large number of matrix optics theories were put forward^200,201^, deriving the HLG mode to describe the controllable generation of a vortex array in the AMC system^76,77^. Similar to the HLG mode, the HIG mode is also a multi-singularity VB, which can be generated in special cavities with selective pumping^80^ and an SLM^81^. Recently, our group proposed a method of tuning the periodic orbits of an SU(2) GM in a degenerate cavity and further tuning the multi-singularity OAM of SU(2) VBs^202–204^, as shown in Fig. 12b. In addition to HIG, HLG and SU(2) VBs, many other multi-singularity VBs with special mathematical formulations were generated with different control methods, such as trochoidal VBs^95^, transverse-mode-locking vortex lattices ^202,203^, and polygonal VBs^96^.

**Fig. 13:**
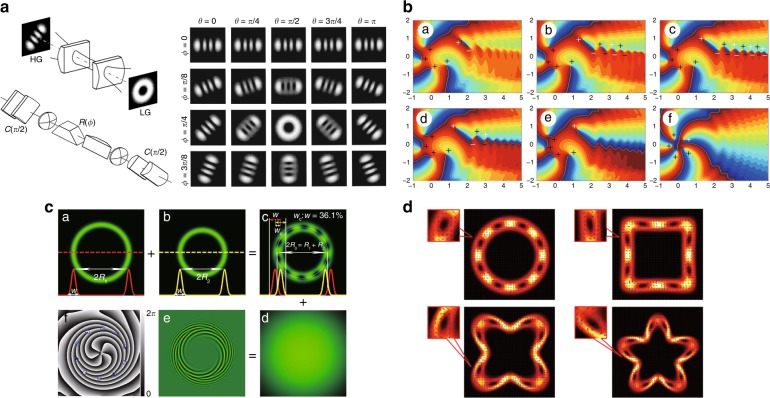
Generation of multi-singularity VBs. **a** Exploring the singularity splitting phenomenon in AMC systems^200^. **b** Multiple singularity formation in fractional OAM VBs^211^. Tailoring multi-singularity beams with **c** a circular vortex array^207^ and **d** an optical vortex array along an arbitrary curvilinear arrangement^208^. **a** Reprinted with permission from ref. ^200^, Copyright (2019), with permission from Elsevier. **b** Reprinted with permission from ref. ^211^, OSA Publishing. **c** Reprinted with permission from ref. ^207^. Copyright (2019) by John Wiley and Sons. **d** Reprinted with permission from ref. ^208^, OSA Publishing

In addition to the above multi-singularity modes, people are pursuing more freely tailored methods for arbitrary singularity distributions. SLM modulation combined with a laser source for on-demand modes has been favoured^205^. Recently, increasing numbers of tailored singularity distributions have been designed and realized via SLMs, such as rectangular and circular multi-singularity arrays^206,207^ and arbitrary curvilinear arrays^208^, and quadrant-separable singularity control^209^, as presented in Fig. 13.

There are still many novel methods of tuning the multi-singularity OAM in more types of exotic OVs. For instance, various OV arrays can be generated by coherent combining technology with digital control^210^. Infinite scalar and vector OV arrays can be realized in fractional OAM VBs^211,212^. On-demand multi-singularity VBs can be generated based on the appropriate combination of optical scattering and discrete rotational symmetries of optical isotropic masks^213^ and can be electrically and optically controlled via anisotropic masks^214,215^.

Despite the numerous multi-singularity manipulation methods, the realization of universal and versatile tunability will be the everlasting target in the future.

## Advanced applications of tunable VBs

### **O**ptical tweezers

Optical tweezers that trap particles using an optical force were proposed by Ashkin^216^, who won the Nobel Prize in 2018. Benefitting from the study of OAM interactions with matter, OVs were first used in 1995 in optical tweezers and extended to the optical spanner^19^, where particles can be trapped and driven to move around the singularity. Then, the transformation from optical OAM to mechanical AM was widely studied^32–34^.

**Fig. 14:**
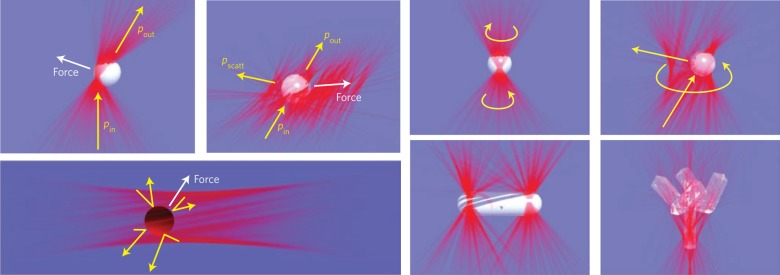
Optical tweezers with VBs can manipulate not only the positions of particles but also their motion, such as precession, nutation, spin, and more complicated orbital motion^217^. Reprinted by permission from Springer Nature: Nature Photonics^217^, Copyright (2019)

**Fig. 15:**
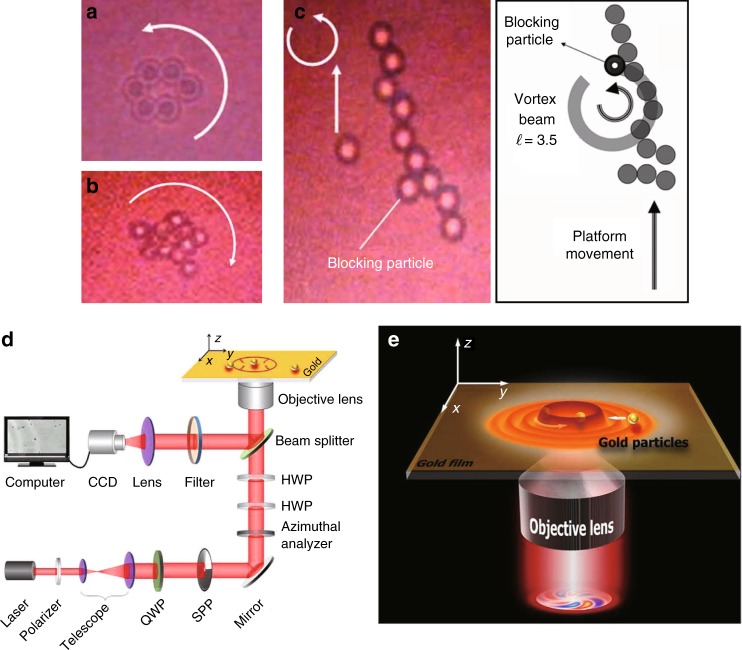
Particle manipulation using OVs. **a**, **b** Manipulating the rotation of multiple particles and **c** aligning and transporting particles with fractional VBs^219^. **d** Setup and **e** schematic of plasmonic vortex tweezers for manipulating metal particles^223^. **a**–**c** Reprinted with permission from ref. ^219^, OSA Publishing

With the improvement of vortex tunability, new-generation tweezers with OVs have shown distinct advantages^34,217^. As demonstrated in Fig. 14, the novel vortex tweezers can conveniently manipulate not only the spatial positions of particles but also the multiple degrees of freedom of particles, largely extending the automated guiding, assembly, and sorting technology^217,218^. With the control of multi-singularity VBs, many new techniques were designed and applied to trap multiple particles^82,217,218^, including the fractional optical VB for optical tweezers^219^. With femtosecond VBs, the tweezers carrying special nonlinear properties can be used to manipulate optical Rayleigh particles^220^. Furthermore, with femtosecond vector VBs, nonlinearity-induced multiplexed optical trapping and manipulation was designed^221^, where the number of traps and their orientations could be flexibly controlled. In addition to dielectric particles, metal particles can also be manipulated by novel plasmonic vortex tweezers^222^, where the vortex field of surface plasmon polaritons can be generated by focusing vector VBs onto a metal film. Plasmonic vortex tweezers as depicted in Fig. 15d, e were shown to be superior in manipulating metal particles with large flexibility^223^.

### **O**ptical communication

**Fig. 16:**
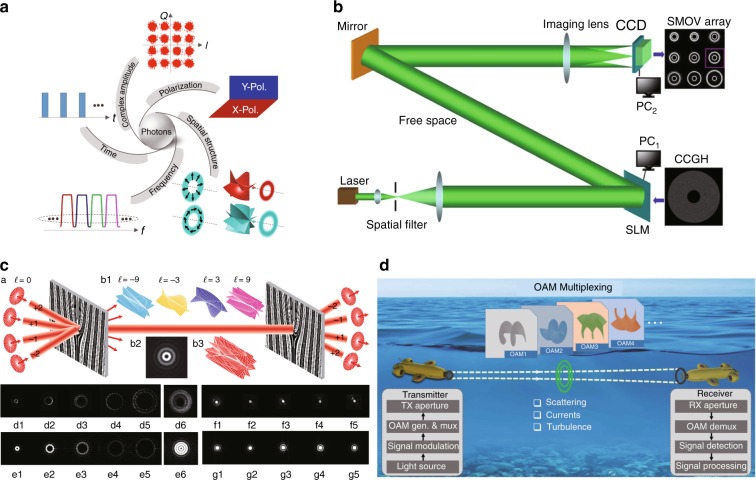
Optical communication using OVs. **a** Optical communication can be realized by modulating the time domain, frequency domain, amplitude, polarization, and OAM^225^. **b** Schematic of sidelobe-modulated OVs for free-space communication^230^. **c** Dammann-grating-enabled OAM multiplexing technology raising the large capacity to the Pbit level^234^. **d** Underwater optical communication using VBs^235^. **b** Reprinted with permission from ref. ^230^, OSA Publishing. **c** Reprinted by permission from Springer Nature: Light: Science & Applications^234^, Copyright (2019)

In addition to the polarization, amplitude, pulse shape, and wavelength of light, the OAM can be used as an alternative degree of freedom for multiplexing modulation, enlarging the capacity of optical communication^39^, which is also referred to as mode/spatial-division multiplexing (MDM/SDM)^224^. Optical communication by OAM multiplexing has enabled breaking the Tbit level^43,44^, much beyond the conventional scheme, thus greatly broadening the application scope^225,226^. With the study of VB propagation in the atmosphere, free-space communication using vortices was gradually improved^227–229^. Furthermore, a sidelobe-modulated OV method was proposed for free-space communication with a significant increase in the data transmission capacity^230^. With the development of multi-singularity-tunable VBs, the capacity and speed of communication can be further improved^231^. A variety of special fibres for OAM mode transmission were designed to enable fibre-based vortex communication technology^232,233^. Recently, a new OAM multiplexing technology using Dammann vortex gratings in fibre-free-space coupled systems realized massive OAM state parallel detection^234^, offering an opportunity to raise the communication capacity to the Pbit level. OAM-multiplexing-based communication was also demonstrated under many extreme circumstances, such as underwater communication^235^ illustrated in Fig. 16d, high-dimensional quantum communication^236^, and long-distance fibre communication^237^.

### Quantum entanglement

With the recent mature quantum descriptions of twisted photons^31^, OAM entanglement has engendered plenty of applications^134^. For instance, high-dimensional quantum key distribution (QKD) protocols can be designed based on mutually unbiased bases related to OAM photons^238^, which motivated high-dimensional quantum cryptography for high-security communication^239^. The quantum memory technology for OAM photonic qubits was recently proposed to provide an essential capability for future networks^240^. Because of the inherent infinite dimension of OAM, the OAM of photons has been successfully used to realize quantum storage in various systems, such as atomic ensembles^241^ and rare-earth-ion-doped crystals^242^, benefiting high-capacity communication. High-dimensional OAM entanglement was also successfully used in high-efficiency digital spiral imaging^243^. Employing the Hong–Ou–Mandel interference of OAM photons, quantum cloning technology for making copies of unknown quantum states was presented^244^. With the development of vector VB manipulation, SAM and OAM were combined for quantum communication to further scale the capacity and speed^245^. Quantum teleportation using OAM can largely improve the technical control of scalable and complicated quantum systems^246^. To date, the entangled photon system with the highest number of qubits (18 qubits with six entangled photons) with OAM as one degree of freedom has been produced^247^. Very recently, as a remarkable breakthrough, quantum entanglement between the SAM and OAM states was realized in a metamaterial^47^.

**Fig. 17:**
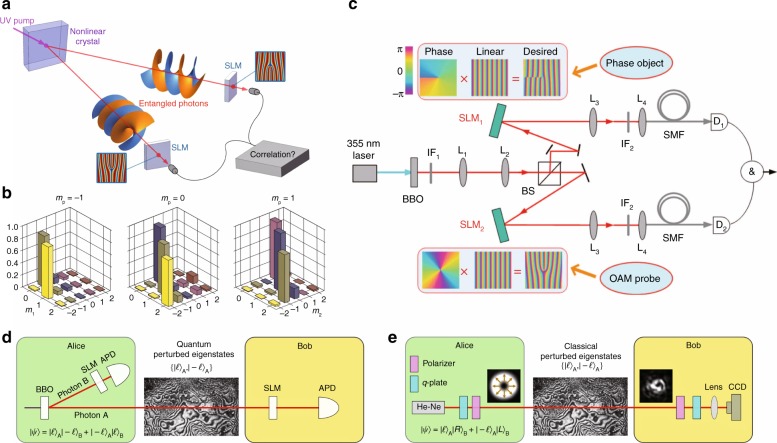
Quantum technologies using OVs. **a** OAM-entanglement photon pairs generated by the spontaneous parametric down conversion process. **b** Quantum tomography experimental data demonstrating that the OAM of the pump beam is transferred to the sum of the OAM of the generated photons (*m*_p_ = *m*_1_ + *m*_2_)^31^. **c** Setup for quantum digital spiral imaging^243^. Comparison between quantum communication using **d** entangled photons and **e** non-separable states of vector OVs^250^. **b** Reprinted by permission from Springer Nature: Nature Physics^31^, Copyright (2019). **c** Reprinted by permission from Springer Nature: Light: Science & Applications^243^, Copyright (2019). **d**, **e** Reprinted by permission from Springer Nature: Nature Physics^250^, Copyright (2019)

In addition to scalar phase OVs, vector polarization OVs also have fruitful quantum properties. The non-separable states between the polarization and space share common properties with the entangled state of photons, which is also called the classical entanglement state^71,248^. The quantum tomography, Bell parameter, concurrence count, and linear entropy can be realized in vector OVs akin to corresponding quantum measurements^148,149,248^. Taking advantage of the high-dimensional properties of the non-separable states, quantum walks can be implemented by vector OV modes of light, enlarging the scalable range^249^. Entanglement beating generated in vector VBs can be used to control spin–orbit coupling in free space^135^. High-dimensional entanglement has also been utilized in coding quantum channels to improve high-capacity optical communication^250^, as illustrated in Fig. 17d, e.

### Nonlinear optics

With the development of high-power and large-energy VBs^92,180,251^, the peak power can exceed the threshold of various nonlinear effects, providing conditions to explore novel nonlinear conversion phenomena related to OAM^48,49,251^. Conventionally, the development of nonlinear optics was based mainly on the scattering that obeys momentum conservation (Rayleigh scattering, Brillouin scattering, Raman scattering, etc.), and the corresponding development of nonlinear frequency transformation effects (frequency doubling, frequency summing, four-wave mixing, etc.) has benefited a myriad of applications. In the new century, new transverse nonlinear transformation effects have been developed based on AM conservation, such as TC variation during the processes of frequency doubling^25,26^, summing and mixing^252,253^, tunable OAM high-harmonic transform^48,49^, and OAM strong-field physics^175^. Recently, these OAM harmonic generations have been widely applied in nanomaterials for the control of nonlinear phases^254^, the Pancharatnam–Berry phase^255^ and beam shaping^256^. In addition, there are many novel physical phenomena coupled with nonlinear OAM-frequency conversion, such as the rotational Doppler effect^257^ and rotational nonlinear absorption^258^.

### Nanotechnology

Due to the rapid development of nanofabrication and increasing demands for nanotechnology applications, nanointegrated on-chip vortex generators have emerged for emitting VBs at the nanoscale, such as integrated silicon-chip-based VB emitters^259^, vortex vertical-cavity surface-emitting lasers (VCSELs)^260^, angular gratings^42^, micro-nano-OAM laser emitters^261^, and various metasurface designs^262^. Taking advantage of nanoscale VBs, many novel phenomena related to OAM in nanophotonic materials have been demonstrated, such as non-dispersive vortices^263^ and SAM-to-OAM conversion effects^46,47^. Combined with new nanomaterials, many vortex-emitting materials and devices with unique functions have been invented, such as vector vortex on-chip generators^264^ and parallel OAM processors^265^. Combining quantum technology and nanotechnology, a photonic chip capable of purifying the OAM quantum states was recently produced, which possesses great potential to develop on-chip quantum calculation^266^.

### Optical machining

Due to the nature of high-order modes, VBs show weaker capability in conventional machining processes, such as laser cutting and laser punching, than the fundamental Gaussian beam. However, in some special applications, vortex light has distinct advantages. When a metal surface is processed by different vector VBs, various intriguing new patterns can be selectively displayed under light illumination^267,268^. Moreover, the surface can exhibit different patterns when the illuminated light has different incidence angles^269^. In addition to the angular sensitivity, a polarization-sensitive surface was fabricated based on a similar technique using vortex processing, i.e., different patterns were exhibited when the surface was illuminated by light with different polarizations^268^. Utilizing nanophotonics technology, nanoscale VBs were used in nanostructure fabrication. For instance, the chiral nanoneedle structure can be easily fabricated by a perpendicular VB through the transfer of the consequential torque from OAM light to the object^173,269,270^. Similar methods can produce some other nanostructures, such as helical surface reliefs^271^ and monocrystalline silicon needles^272^. Recently, high-power ultrashort OAM-tunable VBs were combined with femtosecond laser direct writing technology to process more special structures, such as multi-waveguide^266^ and micro-pipe structures^174^.

### Microscopy and imaging

**Fig. 18:**
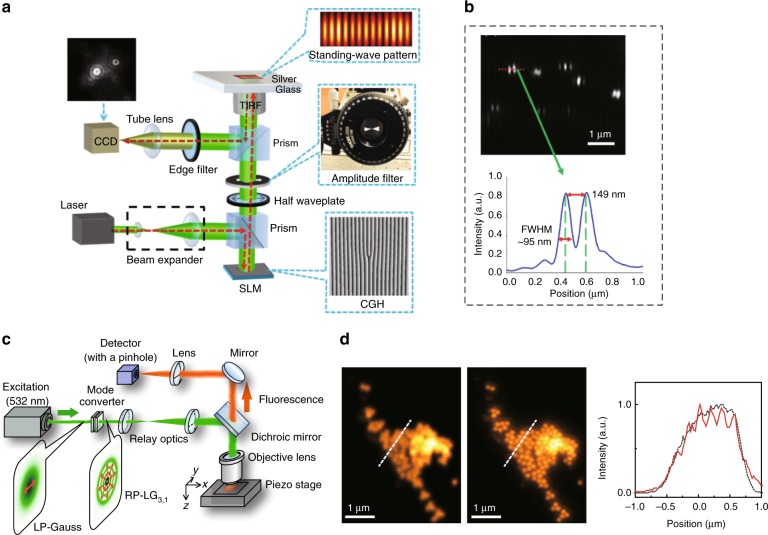
High-resolution imaging using OVs. **a** Setup of the plasmonic structured illumination microscopy technique, and **b** imaging results with super-resolution^277^. **c** Setup of superoscillation focusing imaging using a vector VB, and **d** imaging results with super-resolution^278^. **a**, **b** Reprinted with permission from ref. ^277^, OSA Publishing. **c**, **d** Reprinted with permission from ref. ^278^, OSA Publishing

The unique spiral phase of VBs can be used in phase-contrast microscopy, demonstrating high-resolution micro-imaging^37^. Applying OAM analysis in the imaging method, the novel digital spiral imaging technique was proposed to improve the resolution^273^. Currently, imaging using OAM has already realized super-diffraction-limit resolution^38^. In recent years, a growing number of novel microscopy and imaging technologies using VBs have emerged, reaching increasingly higher resolution. For instance, plasmonic structured illumination microscopy using standing surface plasmon waves induced by OVs was proposed, realizing high-resolution wide-field imaging^274^. This microscopy was further improved by using perfect VBs (VBs with a controllable ring radius) to enhance the excitation efficiency and reduce the background noise^275^. With the development of multi-singularity beams, a vortex array was used to harness the point-spread function to realize high-resolution far-field microscopy^276^. Specifically, fractional VBs were also used for precise microscopy to reach sub-100-nm resolution^277^. With the advanced vector VBs having a special polarization structure, the super-resolution imaging reached an even higher resolution^278^, as shown in Fig. 18c, d. With the quantum properties of VBs, quantum ghost imaging was combined with twisted photons, opening new routes for imaging techniques^243^. As a remarkable breakthrough of microscopy using OVs, the stimulated emission depletion (STED) microscopy technique proposed by Willig et al.^279^, in which the vortex phase is modulated in STED beams to realize super-resolution, was awarded the 2014 Nobel Prize in Chemistry.

### Biomedicine and chemistry

Using OV tweezers, one can manipulate and assemble some proteins and other biomolecules, greatly advancing the development of structural chemistry and biomedical photonics^34,36^. Note that VBs and some organic molecules all have chirality, and the chirality of the vortex phase can interplay with that of a biomolecule, which has promoted a number of applications in biomedicine and chemistry^280^. For instance, VBs can be used to assemble DNA^36^ and resolve enantiomers^281^ due to the chirality coupling effect. By applying this method to chiral metamaterials, novel sensing technology was proposed to detect many enantiomers or biomolecules, such as amino acids, sugars, and nucleotides^282^. Additionally, the functionalities of transporting subcellular organelles and exerting less photodamage on the trapped particle was developed for vortex tweezers, which have been used in sophisticated single-cell nanosurgery^283^. The advanced microscopy brought about by VBs was also used for observing biological cell structures with high resolution^279^. Most recently, vortices were directly generated from organic materials^284^, with further development of organic illumination and chemical detection technologies expected in the future.

### Metrology

**Fig. 19:**
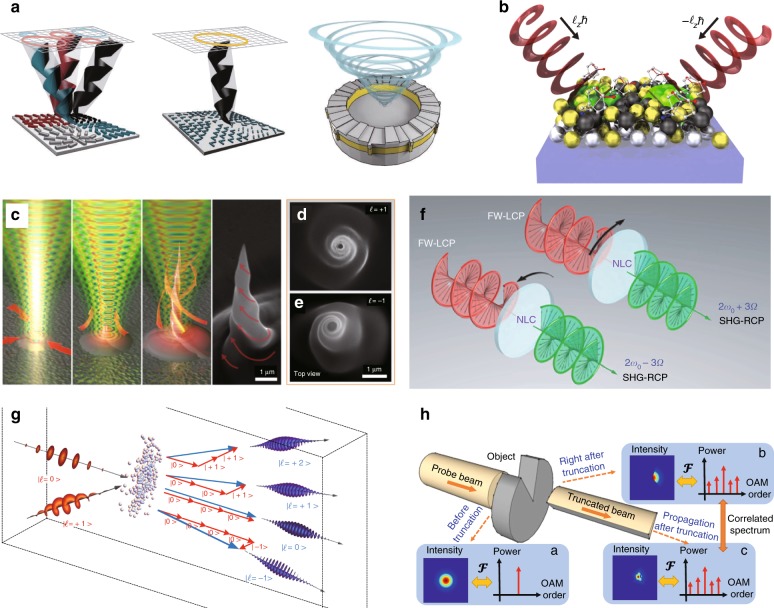
Nanotechnology, nonlinear conversion, and detection using OVs. **a** Various integrated nanoscale vortex emitters^259^. **b** Sorting and resolving enantiomers or molecules with different chiralities^281^. **c** Machining a nanoscale needle using VBs with **d** clockwise rotation and **e** counterclockwise rotation structures^270^. **f** Schematic diagram of the rotational Doppler effect in nonlinear optics^257^. **g** Schematic diagram of the OAM conservation process in the high-harmonic generation of VBs^49^. **h** Schematic diagram of object detection using the OAM spectrum^288^. **a** From ref. ^259^. Reprinted with permission from AAAS. **b** Reprinted/adapted from ref. ^281^. © The Authors, some rights reserved; exclusive licensee American Association for the Advancement of Science. Distributed under a Creative Commons Attribution NonCommercial License 4.0 (CC BY-NC) http://creativecommons.org/licenses/by-nc/4.0/. **c**–**e** Reprinted with permission from ref. ^270^, OSA Publishing. **f** Reprinted by permission from Springer Nature: Nature Physics^257^, Copyright (2019). **g** Reprinted by permission from Springer Nature: Nature Communications^49^, Copyright (2019). **h** Reprinted with permission from ref. ^288^, OSA Publishing

Based on the light-matter interaction through which the OAM of light can be coupled with the mechanical momentum, VBs can be used to detect object motion, including spin motion^285^ and lateral motion^286^. With recent advances in nanophotonics and nanofabrication, the precision of detection has reached the nanoscale, and VBs can be used for label-free single-molecule detection in metamaterials^287^. Recently, the OAM spectrum, acting as a new powerful tool, was used in optical detection, in which the difference between the OAM spectra of incident and outgoing light revealed the topography of the target^288^, as depicted in Fig. 19h. Similar OAM-spectrum methods have been successfully applied to detect complicated turbulence in the atmosphere^289^ and ocean^290^. Recently, with the study of the interaction between OVs and plasmonic nanoslits^291^, VBs have been used to detect the nanostructure on metal films, opening the door for on­chip compact OAM detection^292^. There are also several devices and structures for detecting OAM states. For instance, a virtual rotational antenna structure was designed to generate the rotational Doppler effect, and the signal of the Doppler shift could be detected to reveal the OAM of the corresponding OV^293^. The on-chip plasmonic nanoslit structure can produce different scattering effects for OVs with different TCs, serving as a useful tool for the discrimination of OAM^294^. Moreover, some on-demand metasurface^262^ and liquid crystal^170,171,265^ devices have shown great potential for detecting OAM, enabling the further development of precise metrology technologies.

### Astronomy

**Fig. 20:**
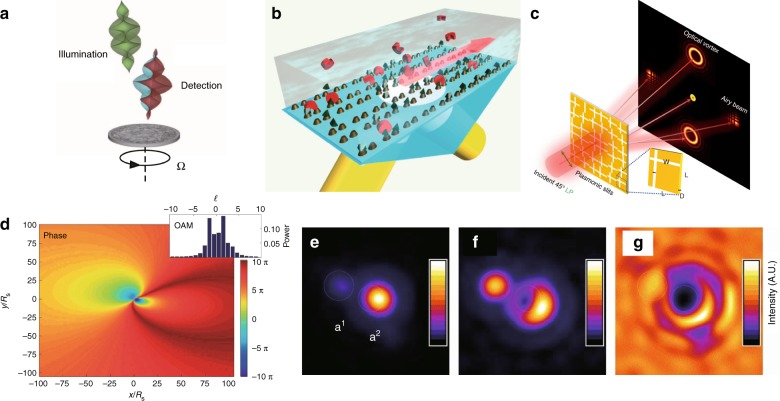
Metrology and astronomy using OVs. **a** VBs can be used to detect the spin motion of an object^285^. **b** Twisted light can be used for label-free single-molecule detection^287^. **c** VBs can be used with plasmonic nanoslit structures for multi-channel OAM generation and detection^291^. **d** Vortex generation around a rotating black hole^302^. **e**–**g** Imaging of an OV coronagraph^297^. **a** From ref. ^285^. Reprinted with permission from AAAS. **b** Reprinted by permission from Springer Nature: Nature Materials^287^, Copyright (2019). **c** Reprinted with permission from ref. 291. Copyright (2019) by John Wiley and Sons. **d** Reprinted by permission from Springer Nature: Nature Physics^302^, Copyright (2019). **e**–**g** Reprinted with permission from ref. ^297^, OSA Publishing

OVs not only have been artificially created in laser beams but also naturally exist in the cosmic microwave background^35^. In 2003, Harwit described astrophysical processes of OAM light generation, including photon scattering and vortex generation in the environments surrounding energetic sources, e.g., masers, pulsars, and quasars^35^. To make an astronomical survey that took advantage of OVs, an OV coronagraph was designed^295^ and experimentally verified^296^ by Swartzlander’s group, which has made many breakthroughs in astronomical demonstration^297^. In addition to the scalar vortex masks used in these coronagraph devices, vectorial masks were also implemented in coronagraphs at nearly the same time as Swartzlander’s work in 2005^298^. With the development of vector OVs, the vortex coronagraph implemented in international ground-based telescope facilities has been based on vectorial vortex masks to obtain higher sensitivity and lower aberrations^299^. With the recent development of multi-singularity tunability, adaptive multiple-vortex coronagraph masks have been developed for multiple-star detections^300,301^. In 2011, Tamburini et al.^302^ reported the OAM light effect around rotating black holes, which provided a new method to detect black holes, as shown in Fig. 20d. Interestingly, astronomical applications are always accompanied by sci-fi themes, and vortex light has been declared to be a fast, furious and perfect tool for talking to aliens and detecting alien civilizations due to its unique properties^303^.

### Other advances

OVs indeed demonstrate various characteristics, not only as VBs analysed under the paraxial approximation but also as a general spatial singular field with fractality of singularities. In addition, OVs are not restricted to linear space but have been extensively studied in nonlinear media in connection with optical solitons^7,22–24^. Moreover, topological vortex waves can be studied in other spectra in addition to the light field, such as microwave vortices^304^, acoustic vortices^305^ and X-ray vortices^50^. Vortex electron beams^59^ and neutron beams^60^ with unique OAM properties were also produced and investigated. Very recently, gravitational waves with AM were observed and could be used for trapping and guiding cosmic bodies^306^. Overall, there are currently numerous promising and amazing applications related to OVs with unlimited possibilities that require further exploration.

## Conclusions and perspectives

This review article is dedicated to commemorating the 30th anniversary of the birth of OVs, covering the development history from fundamental theories to tunable vortex techniques and then to widespread scientific applications. We first reviewed the theoretical foundation of OVs and emphasized the unique properties related to OAM, TC, and singularities. Then, we reviewed the recent advances in tunable VBs, where the tunability includes not only wavelength tunability and temporal tunability but also OAM tunability. Recent vortex generation methods with different kinds of tunability were reviewed, revealing the development of optical field manipulation. Taking advantage of the advanced vortex manipulation techniques, widespread novel applications have boomed in the new century. We reviewed the various applications in different branches of science as comprehensively as possible. The development tendency of OVs is a typical example that theories guide new applications and that application demands inspire new theories. To date, OVs are still hot topics and have high potential for both theories and applications.
